# Identification of Corn Peptides with Alcohol Dehydrogenase Activating Activity Absorbed by Caco-2 Cell Monolayers

**DOI:** 10.3390/molecules29071523

**Published:** 2024-03-28

**Authors:** Zhe Wang, Guanlong Li, Xiaolan Liu

**Affiliations:** 1 College of Food Engineering, Harbin University of Commerce, Harbin 150076, China; wangzhe@qqhru.edu.cn; 2 Key Laboratory of Corn Deep Processing Theory and Technology of Heilongjiang Province, College of Food and Bioengineering, Qiqihar University, Qiqihar 161006, China; 03580@qqhru.edu.cn

**Keywords:** ADH activating peptides, gastrointestinal digestion, Caco-2 cell monolayer, corn, identification

## Abstract

Alcohol dehydrogenase (ADH) plays a pivotal role in constraining alcohol metabolism. Assessing the ADH-activating activity in vitro can provide insight into the capacity to accelerate ethanol metabolism in vivo. In this study, ADH-activating peptides were prepared from corn protein meal (CGM) using enzymatic hydrolysis, and these peptides were subsequently identified following simulated gastrointestinal digestion and their absorption through the Caco-2 cell monolayer membrane. The current investigation revealed that corn protein hydrolysate hydrolyzed using alcalase exhibited the highest ADH activation capability, maintaining an ADH activation rate of 52.93 ± 2.07% following simulated gastrointestinal digestion in vitro. After absorption through the Caco-2 cell monolayer membrane, ADH-activating peptides were identified. Among them, SSNCQPF, TGCPVLQ, and QPQQPW were validated to possess strong ADH activation activity, with EC_50_ values of 1.35 ± 0.22 mM, 2.26 ± 0.16 mM, and 2.73 ± 0.13 mM, respectively. Molecular Docking revealed that the activation of ADH occurred via the formation of a stable complex between the peptide and the active center of ADH by hydrogen bonds and hydrophobic interactions. The results of this study also suggest that corn protein hydrolysate could be a novel functional dietary element that helps protects the liver from damage caused by alcohol and aids in alcohol metabolism.

## 1. Introduction

China’s corn production reached 288 million tons in 2023 [1], solidifying its position as one of the world’s major corn-producing regions. In addition to being staple meal and an essential part of animal feed, corn can also be used as a raw material to make alcohol, corn oil, and corn starch [2,3,4,5]. Corn protein meal (CGM), a byproduct of wet milling of corn starch, contains approximately 60% protein [6]. The CGM faces challenges due to poor solubility and strong hydrophobicity, limiting its effective utilization in the food industry. By modifying CGM to create biologically active products, not only can the structure of corn industry be optimized, but it can also expand the corn industrial chain and enhance product value. This endeavor holds significant promise for the deep processing of CGM, offering a vital solution to agricultural challenges and contributing to the resolution of the “three dimensional rural issues”.

The liver plays the key role in metabolizing alcohol within the human body, responsible for metabolizing 80–90% of alcohol through the alcohol dehydrogenase (ADH) pathway [7]. ADH (EC 1.1.1.1) is a zinc-containing metalloprotein enzyme that utilizes nicotinamide adenine dinucleotide (NAD^+^) as a co-factor to convert alcohol into the toxic acetaldehyde, which is subsequently oxidized into non-toxic acetic acid with aldehyde dehydrogenase (ALDH) [8,9]. The activity of ADH in the liver is a major determinant of alcohol metabolism [10]. Short-term excessive alcohol consumption diminishes ADH activity in the liver, leading to the accumulation of excessive alcohol and contributing to the development of alcoholic liver disease (ALD), a significant global public health concern. Recent years have seen a rise in ethanol consumption, correlating with an increase in alcohol-related health issues, accounting for approximately 1/20 of global mortality [11]. In China, an estimated 62 million individuals suffer from alcohol-induced liver damage, with ALD being the leading cause of liver-related mortality [12]. While moderating alcohol consumption is an effective approach to prevent ethanol-induced liver damage, the identification of new bioactive agents for the prevention and treatment of ALD remains crucial.

Bioactive peptides, deemed natural agents promoting human health, demonstrate diverse biological activities. Besides nutritional value, they also exhibit antioxidative, hypotensive, anti-inflammatory, antimicrobial, and hepatoprotective properties, etc. [13,14,15,16]. Studies have reported that peptides derived from diverse sources, including corn [17,18], black soybeans [19], clam [20], chickpeas [21], and chicken [22], may enhance alcohol metabolism. Due to the need for bioactive peptides to exert their biological activity in the body, they must overcome two important physiological barriers: the complex enzymatic degradation in the gastrointestinal tract, and the low permeability of the intestinal epithelium. This process enables them to be absorbed in their intact form and to reach their target sites via the bloodstream [23]. Therefore, this study aims to isolate ADH-activating peptides from CGM and identify them after simulated gastrointestinal digestion and absorption through the Caco-2 cell monolayer membrane.

## 2. Results and Discussion

### 2.1. Screening for Optimal Enzymatic Conditions

**Figure 1:**
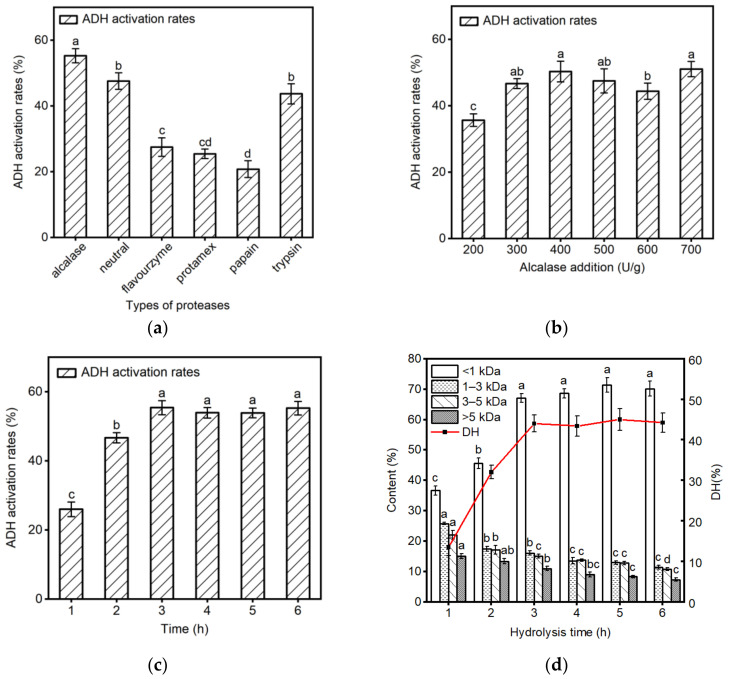
Screening for optimal enzymatic conditions. (**a**) ADH activation activity of CPHs prepared using different proteases; (**b**) ADH activation activity of hydrolysates with different alcalase addition; (**c**) ADH activation activity of hydrolysates with different hydrolysis time using alcalase; (**d**) DH and MW distribution of hydrolysates with different hydrolysis time using alcalase. Data are expressed as the mean ± SD, and differences were analyzed using Tukey’s test, *n* = 3. The results without common superscript letters (a–d) were statistically different (*p* < 0.05).

CGM is a major byproduct generated during the wet milling process in the corn starch industry. It contains 60% protein, of which approximately 65% is zein and 30% is glutelin. Additionally, it is comprised of about 30% carbohydrates, with around 15% being starch and the remaining consisting of fats and fiber [24]. After the removal of starch by *α*-amylase, the protein content of corn protein was 87%. Then, the corn protein powder was prepared into a 5% suspension and subjected to enzymatic hydrolysis using six distinct proteases (alcalase, neutral, flavourzyme, protamex, papain, and trypsin). The effect of hydrolysates was assessed using molecular weight (MW) arrangement, degree of hydrolysis (DH), and ADH activation activity. As shown in Figure 1a, among these six enzymes, the corn protein hydrolysates produced by alcalase hydrolysis exhibited the highest ADH activation activity (55.2 ± 2.15%) at the concentration of 1 mg/mL, followed by neutral protease and trypsin. Peptides with varying degrees of activity may be produced as a result of unique enzyme-specific cleavage sites [25]. Xiao et al. [26] investigated the impact of chicken hydrolysates on ADH stability, and alcalase was also used to hydrolyze together with further bioactivity-oriented isolation and identification. The results suggested that ADH activation rate of corn protein hydrolysates (CPHs) obtained by alcalase was significantly higher than that of other five proteases.

Further investigation was conducted on the influence of alcalase addition on the ADH activation activity of CPHs. As shown in Figure 1b, at an alcalase addition of 400 U/g, the ADH activation rate of CPHs reached its maximum (50.3 ± 3.12%), and with an increase in enzyme addition, the activation rate of CPHs did not show a significant increase (*p* < 0.05). Therefore, the enzyme addition of 400 U/g was selected as the enzyme digestion condition of alcalase.

Subsequently, the impact of hydrolysis time of alcalase at an enzyme addition of 400 U/g on the ADH activation activity of CPHs was optimized. According to Figure 1c, after hydrolysis for three hours, the ADH activation activity reached its peak (55.4 ± 2.03%), and further extension of the hydrolysis time did not result in a significant increase (*p* < 0.05) in ADH activation activity. This observation suggests that within the initial 3 h period, the proteins were effectively hydrolyzed into peptides exhibiting maximal ADH activation activity.

To investigate the hydrolysis efficiency of alcalase on corn protein and to confirm the inference aforementioned, the DH and MW distribution of the hydrolysis products were determined at different hydrolysis times. As depicted in Figure 1d, the DH continuously increased with prolonged hydrolysis time. When the hydrolysis time reached 3 h, the degree of hydrolysis stabilized. Zheng et al. [27] reported a similar DH trend of corn glutelin. Likewise, with the extension of the hydrolysis time, the content of small peptides (<1 kDa) gradually increased, while the content of 1–3 kDa, 3–5 kDa, and >5 kDa components decreased gradually, stabilizing after 3 h of hydrolysis. This indicates that peptide components of <1 kDa may have higher ADH activation activity. Sonklin et al. [28] similarly found that peptide components of <1 kDa exhibit higher antioxidant activity. Considering the above conclusions, it is determined that using 400 U/g alcalase for 3 h of hydrolysis is the optimal condition for preparing ADH activating peptides.

### 2.2. Separation of ADH Activating Peptides by Ultrafiltration and RP-HPLC

**Figure 2:**
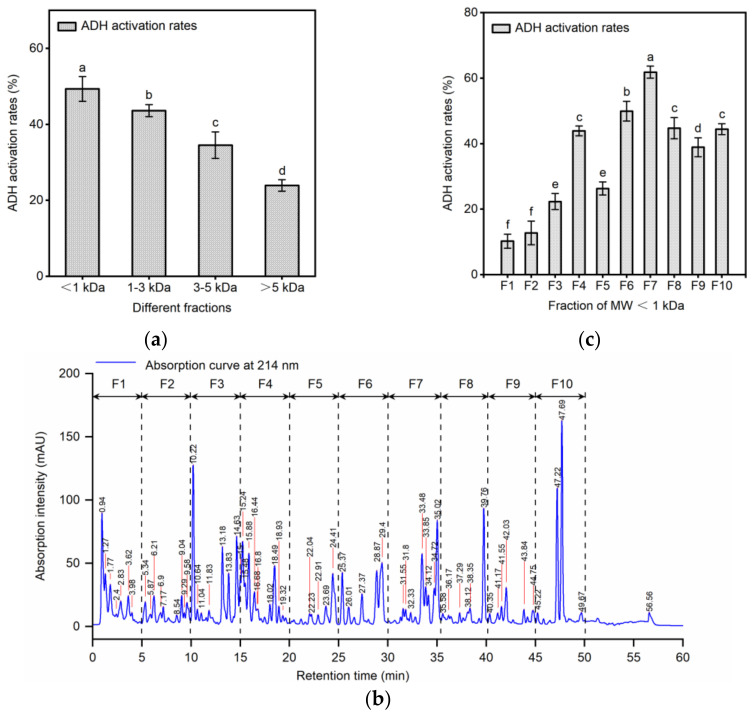
Separation of ADH-activating peptides by ultrafiltration and RP-HPLC. (**a**) The activation of ADH in various ultrafiltration fractions; (**b**) RP-HPLC chromatogram of pep-tides with MW < 1 kDa; (**c**) ADH activation activity of further isolated fractions with MW < 1 kDa (F1 to F10). Different letters above the error bar indicate significant differences. (*p* < 0.05).

The ADH-activating peptides were separated using ultrafiltration and RP-HPLC. Figure 2a shows ADH activation rates of the ultrafiltrated fractions. The MW of those four fractions were MW > 5 kDa, 3 kDa < MW < 5 kDa, 1 kDa < MW < 3 kDa, and MW < 1 kDa, respectively. The result demonstrated that the maximum ADH activity was seen in peptide fraction smaller than 1 kDa. With an increase in the molecular weight range, the associated ADH activation activity decreased. Due to their relative small size and flexibility in fitting into the three-dimensional structure of enzymes, small peptides may have better access to the active site of an enzyme [29]. Shi et al. [30] found that the ability of low molecular weight mushroom peptides (0–3 kDa) to activate ADH was higher than that of high molecular weight components (3–10 kDa). Zhao et al. [31] also reported the same result from mushroom foot peptides. Our findings were in agreement with these previous reports. Therefore, the MW < 1 kDa fraction was selected for further study due to its potent ADH activation activity.

RP-HPLC is a frequently employed technique for peptide isolation and separation, offering insights into variations in hydrophobicity between fractions [32]. The fraction with MW less than 1 kDa was subsequently divided into ten fractions (F1–F10) using RP-HPLC (Figure 2b), and the results are illustrated in Figure 2c. The ADH activation rates of each fraction at the weight concentration of 1 mg/mL is exhibited in Figure 2b. It can be seen that F7 showed the highest ADH activation activity among all the fractions. The ADH activation activity of F7 was measured at 61.82 ± 1.83%, approximately four times higher than the lowest value (F1) and 20% higher than the second-highest level (F6). In reverse-phase chromatography, components with later elution times exhibit stronger hydrophobicity. Xiao et al. [26] utilized RP-HPLC to fractionate the ADH activating peptides, observing that fractions with a later elution time, exhibited greater ADH activation. Hence, F7 derived from MW < 1 kDa was selected for further analysis.

### 2.3. Stability of F7 in Gastrointestinal Digestion

**Figure 3:**
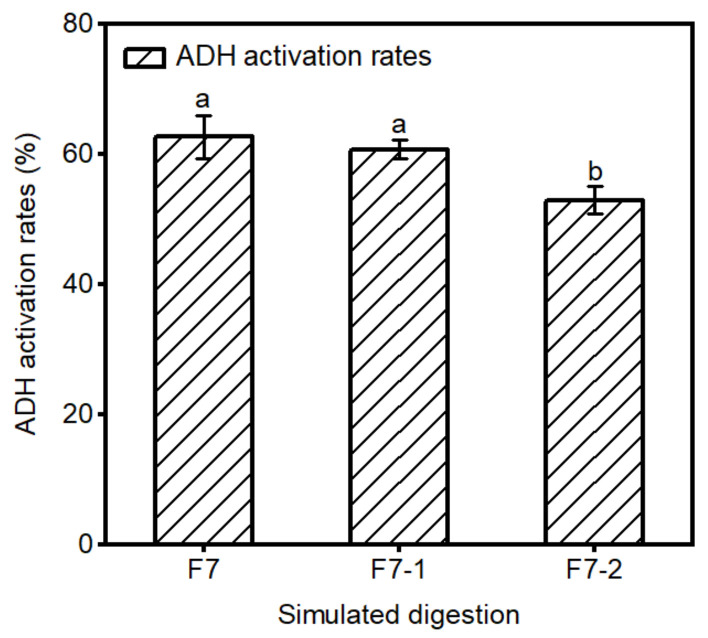
Effect of gastrointestinal digestion on the activity of ADH activating peptides. F7-1 represents the product after pepsin digestion; F7-2 represents the product further digested by trypsin. Different letters above the error bar indicate significant differences. (*p* < 0.05).

Bioactive peptides need to be digested through the gastrointestinal tract and absorbed by the body in the form of active molecules before they can reach the target organs and exert physiological activity [33]. The rates of ADH activation ability of F7 after in vitro digestive stimulation was depicted in Figure 3. The results indicated no significant alteration in ADH activation activity following 90 min of pepsin digestion (F7-1). Subsequently, after an additional 4 h digestion with trypsin, the activity exhibited a significant decrease of 7.80% (F7-2). This result may be attributed to the insensitivity of smaller molecular weight peptides to pepsin, thereby allowing the preservation of active peptide activity [34]. Conversely, trypsin’s action in reducing the overall hydrophobicity of the protein hydrolysis product led to a change in biological activity, consequently resulting in decreased ADH activation activity [35].

### 2.4. Identification of Peptides Absorbed by Caco-2 Cells and Molecular Docking

**Figure 4:**
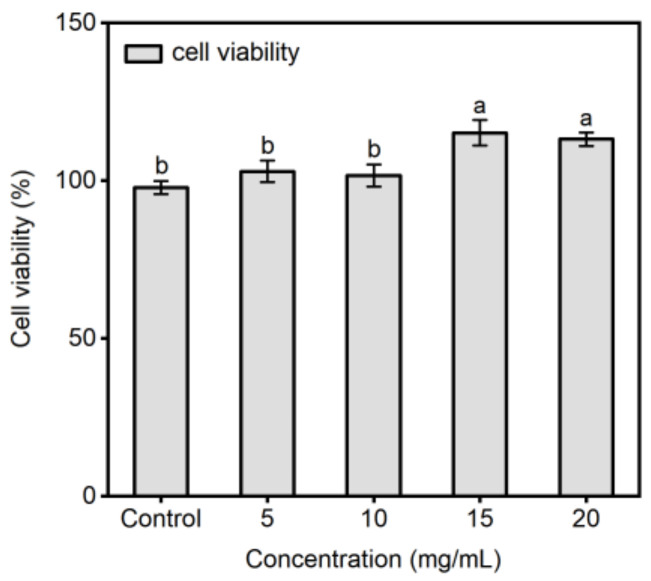
Viability of Caco-2 cells at different concentrations of corn peptide F7-2. Different letters above the error bar indicate significant differences. (*p* < 0.05).

The human small intestinal epithelium consists of numerous well-differentiated and polarized epithelial cells with tight junctions. This epithelium serves as a crucial physiological barrier against the external environment, and is primarily responsible for nutrient absorption [36]. The structure and function of Caco-2 cells are similar to differentiated intestinal epithelial cells, and Caco-2 cell monolayers are commonly used as an in vitro absorption model [37]. Following simulated gastrointestinal digestion, a portion of F7-2 was subjected to absorption experiments using Caco-2 monolayer membrane. Cytotoxicity of the F7-2 corn peptides was shown in Figure 4. At concentrations of 5–20 mg, F7-2 exhibited no cytotoxic effects on Caco-2 cells; instead, it demonstrated a significant proliferative impact at 15 and 20 mg/mL. For this reason, and to ensure the efficiency of doing Caco-2 absorption experiments, A concentration of 10 mg/mL was chosen for the absorption tests. Corn peptides (F7-2) were added to the AP side of Caco-2 cell monolayers and incubated for 2 h. The peptides absorbed in the BL side of Caco-2 cell monolayers were collected and identified using a Q Exactive MS/MS with F7 as the control. There were 13 peptide sequences (shown in Table 1) identified in both the F7 and the BL side, indicating that these peptides could not only withstand gastrointestinal digestion, but also endure cleavage by various brush border membrane peptidases, and finally can be transported intact across Caco-2 cell monolayers.

Molecular docking is a crucial tool for comprehending the structural molecular biology of ligand–biomolecule interactions and for simultaneously predicting the prominent binding modes [38]. To explore the binding mechanism of ADH-activating peptides to ADH receptors, molecular docking was performed with Autodock Vina. As shown in Table 1, 10 out of the 13 peptides that were fully absorbed by Caco-2 monolayers exhibited negative binding energy with ADH through molecular docking, of which TGCPVLQ, SSNCQPF, and QPQQPW had relatively lower binding energy compared to other peptides. A lower binding energy indicates that the peptide is more stable within the active pocket of ADH [39].

**Figure 5:**
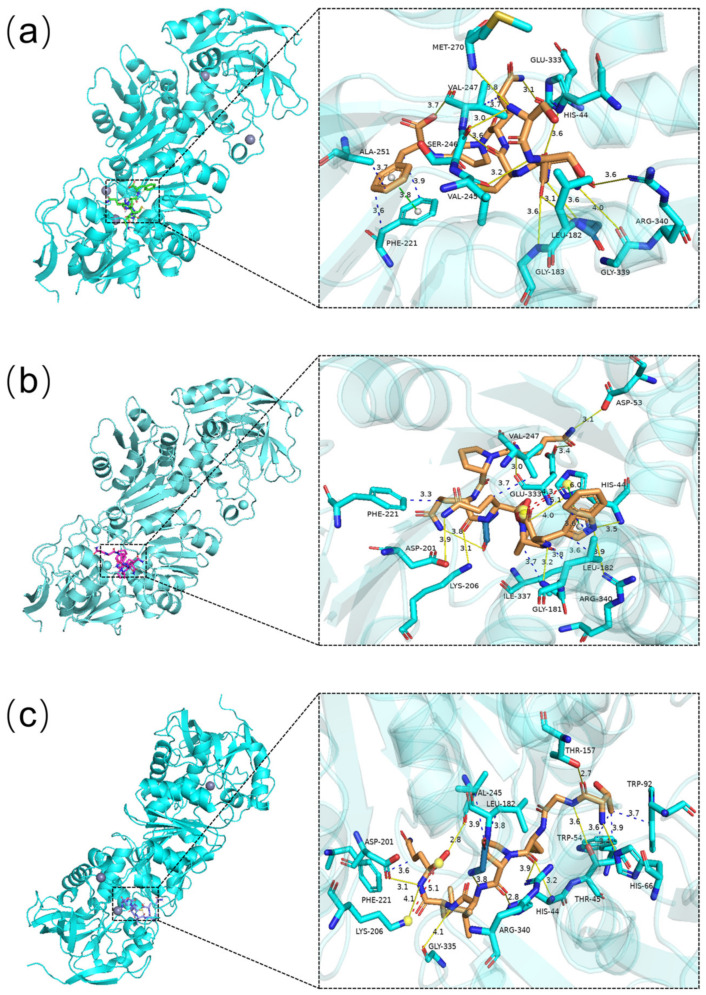
Molecular docking result of three peptides. ADH was selected as receptor and ligands were (**a**) SSNCQPF, (**b**) QPQQPW, and (**c**) TGCPVLQ. Hydrogen bonds, hydrophobic interactions, π-cation interactions, and salt bridges are indicated in yellow, blue, green, and red, respectively.

The results of molecular docking were visualized by selecting three peptides with the lowest binding energy, as depicted in Figure 5. The detailed information of the three peptides combined with ADH were shown in Table 2. Three peptides successfully docked onto the hydrophobic cavity near the ADH active site, primarily binding to ADH through hydrogen bonds and hydrophobic interactions. Twelve hydrogen bonds were formed between SSNCQPF and ADH residues (His 44, Gly 181, Leu 182, Gly 183, Val 245, Ser 246, Val 247, Met 270, Glu 333, Gly 339, Arg 340). Ten hydrogen bonds were formed between QPQQPW and ADH residues (His 44, Asp 53, Gly 181, Asp 201, Lys 206, Glu 333, Gly 335, Arg 340). Eleven hydrogen bonds were formed between TGCPVLQ and ADH residues (His 44, Thr 45, His 66, Thr 157, Gly 181, Asp 201, Lys 206, Val 245, Gly 335, Arg 340). Especially, The aromatic ring in the Phe of SSNCQPF formed a π-stack with Phe 221. QPQQPW formed salt bridges and π-cation interactions with His 44 in ADH. TGCPVLQ established salt bridge with Lys 206 in ADH. The demonstration of all these binding sites suggests that these three peptides bind well to ADH. Xiao et al. [40] reported the binding energy between peptide KPC and ADH enzyme was −6.6 kcal/mol. In this regard, the three peptides identified in this study showed relatively strong interactions with ADH.

### 2.5. In Vitro Activity Verification of Peptides

The three aforementioned peptides, which possess potential to activate ADH, were synthesized and subsequently evaluated for their in vitro activating activity, as detailed in Table 3. Three peptides with low binding energy, as confirmed using molecular docking, all exhibit ADH activating activity. The EC_50_ values were 1.35 ± 0.22 mM (SSNCQPF), 2.26 ± 0.16 mM (TGCPVLQ), and 2.73 ± 0.13 mM (QPQQPW), which were slightly lower than that reported in the previous literature [21], indicating a slightly more potent effect on ADH. This is consistent with earlier results of molecular docking binding energy, showing that the lower the molecular docking binding energy, the lower the EC_50_ value. Therefore, SSNCQPF, TGCPVLQ, and QPQQPW formed stable complexes with the active center of ADH though combined multiple chemical bonds, which consequently activated the enzymatic function of ADH.

## 3. Materials and Methods

### 3.1. Materials and Chemicals

Corn gluten meal (CGM) was manufactured by FuFeng Development Co., Ltd. with a recorded total protein content of 66.56% (*w*/*w*) (Qiqihar, Heilongjiang, China). Alcalase (23,000 U/g), Neutral (21,000 U/mL), flavourzyme (20,000 U/g), Protamex (38,000 U/g), Papain (11,000 U/g) and Trypsin (21,000 U/g) were obtained from Novo Nordisk (Bagsvaerd, Denmark). Caco-2 cells were purchased from JianCheng Co., Ltd. (Nanjing, Jiangsu, China). Fetal bovine serum (FBS) were bought from GE Healthcare (Pittsburgh, PA, USA). Dulbecco’s modified eagle medium (DMEM), Penicillin-streptomycin, and phosphate-buffered saline (PBS) were bought from Gibco U.S. Biotechnology Co. (Grand Island, NY, USA). Ultrafiltion membranes were bought from Pall (Port Washington, NY, USA).

### 3.2. Removal of Starch from CGM

Starch was extracted from the CGM using predetermined protocols [24]. The CGM was immersed in distilled water (10%, *w*/*v*), and 0.1 M HCl was used to bring the pH down to 6.5. Then, α-amylase (30 U/g of protein) was added, and the mixture was incubated at 65 °C for 120 min. Subsequently, the mixture was centrifuged at 4000 r/min for 15 min after being heated in boiling water for 15 min to deactivate the enzyme. Three rounds of distilled water washings were performed on the resultant precipitate. After drying, the pretreatment CGM was obtained and ready for the preparation of corn protein hydrolysates.

### 3.3. Preparation of Maize Protein Hydrolysates with ADH-Activating Activity

The corn protein, from which starch has been removed, was suspended in water to prepare a 5% (*w*/*v*, protein/water) suspension. It was adjusted to the optimal pH for each enzyme using 0.1 M HCl. The hydrolysis was carried out at the optimal temperature and pH for each protease (Table 4), with an enzyme/substrate ratio of 400 U/g and a reaction time of 240 min. After the hydrolysis reaction, the hydrolysate mixture was heated in a boiling water bath for 15 min to terminate the reaction, followed by centrifugation at 4000 r/min for 15 min. The hydrolysis products were collected and freeze-dried for use in subsequent experiments and tests.

Based on the results of the optimal enzyme experiments, a suitable enzyme was selected to study the effect of enzyme addition and hydrolysis time on the ADH activation activity of maize protein hydrolysates. The experimental range of enzyme additions were 200, 300, 400, 500, 600, and 700 U/g (by protein base), the substrate concentration was 5%, and the hydrolysis time was 4 h. Other hydrolysis conditions were selected according to the results of the optimal enzyme experiments. After optimizing the optimal enzyme addition, experiments on enzyme digestion time were performed. The hydrolysis times were 1, 2, 3, 4, 5, and 6 h, and other hydrolysis conditions were as above. Following hydrolysis, the enzyme was deactivated in a boiling water bath for 15 min. The supernatant obtained after centrifugation at 4000 r/min for 15 min was freeze-dried and utilized to assess the ADH activation activity, aiming to determine the enzymatic digestion time.

### 3.4. Separation and Purification of ADH-Activating Peptides

#### 3.4.1. Ultrafiltration

Peptides collected from the optimal enzymatic hydrolysis conditions were ultrafiltered sequentially using ultrafiltration centrifuge tube with 5 kDa, 3 kDa, and 1 kDa. All recovered fractions (MW > 5 kDa; 3 kDa < MW < 5 kDa; 1 kDa < MW < 3 kDa; and MW < 1 kDa) were freeze-dried and stored at −80 °C for use.

#### 3.4.2. High-Performance Liquid Chromatography in Reverse Phase (RP-HPLC)

The fraction with the highest ADH-activating activity were separated using semi-preparative HPLC instrument equipped with a reversed-phase C18 column (Hypersil GOLD PREP C18 column; 5 μm,150 mm × 10 mm). The gradient elution was carried out at a flow rate of 1 mL/min with 20 mmol/L ammonium formate in deionized water (pH = 8, corrected with ammonium hydroxide) as elution A and 20 mmol/L ammonium formate in 75% aqueous acetonitrile solution as elution B. Elution conditions were as follows: 0–10 min, 5% B; 10–50 min, 5–100% B; and 50–60 min return to initial conditions. The column temperature was maintained at 30 °C. The elution was collected every 5 min measured at 214 nm. Ultimately, 10 fractions (F1–F10) were detected, and their ADH activating activity were detected as described later.

### 3.5. Determination of ADH Activation Activity In Vitro

The ADH activation activity in vitro was detected using an improved assay kit method. The specific steps are as follows: 50 μL of the sample solution was mixed with 150 μL of the assay reagent (including buffer, NAD^+^, and ethanol); after equilibrating at 37 °C for 5 min, 50 μL of ADH (0.2 U/mL) was added to initiate the reaction. The absorbance at 340 nm was measured using a Varioskan Flash (Thermo Fisher Scientific, Waltham, MA, USA) full-wavelength scanning multimode reader, with readings taken every 10 s for 10 min. Distilled water was used as a negative control in place of the sample. The reaction kinetics curve was fitted, and the first derivative of the curve at 0 min was calculated as the initial reaction rate. The initial reaction rate of the sample was recorded as V_s_, while that of the negative control was recorded as V_0_. The ADH activation rate of the sample can be calculated using the following equation:

(1) $$ADH\;activation\;activity\;\left(\%\right)=\frac{V_{S}-V_{0}}{V_{0}}\times 100$$

### 3.6. Simulated Gastrointestinal Digestion In Vitro

The corn active peptide was dissolved in distilled water (3%, *w*/*v*), adjusted to pH = 2.0 with 1 mol/L HCl, and then pepsin (enzyme activity: 3000 units/mg) was added at an enzyme-to-substrate ratio of 1:50 (*w*/*w*). The mixture was incubated in a constant temperature oscillator at 37 °C for 90 min to simulate gastric digestion. Subsequently, the pH was adjusted to 7.0 with 1 mol/L NaOH, pancreatin (enzyme activity: 300 units/mg) was added at an enzyme-to-substrate ratio of 1:50 (*w*/*w*), and the mixture was incubated at 37 °C for 4 h to simulate intestinal digestion. After digestion, the sample was kept in a boiling water bath for 10 min to deactivate the enzymes and then cooled to room temperature. The hydrolysate was centrifuged at 4000 r/min for 10 min, the supernatant was collected, and the solution was freeze-dried under vacuum at −20 °C. The sample was resuspended to 1 mg/mL, and its ADH activation activity was determined.

### 3.7. Transport Experiment

#### 3.7.1. Cell Culture

Caco-2 cells were cultured in DMEM supplemented with 10% fetal bovine serum, 1% nonessential amino acid solution, and 1% penicillin-streptomycin solution (10,000 units/mL penicillin and 10,000 μg/mL streptomycin) at 37 °C in an atmosphere of 5% CO₂ and 90% relative humidity. Stock cultures were cultured in 75 cm^2^ tissue culture flasks and sub-cultured at 80% to 90% confluency using 0.25% trypsin and 0.02% EDTA solution. Cells from passages 20–30 were suspended at a density of 2 × 10^5^ cells/mL and added to the apical (AP) side of a 12-well Transwell plate (12 mm diameter, pore size 0.4 μm, growth surface area 1.12 cm^2^, Corning Inc., Corning, NY, USA). The cell culture medium on both the apical and basolateral sides was initially replaced every 2 days for the first week, and subsequently replaced daily. The integrity of the cell monolayer was assessed by measuring transepithelial electrical resistance (TEER) using a Millicell-ERS-2 system (Millipore, Billerica, MA, USA). Only Caco-2 cell monolayers with TEER values exceeding 300 Ω·cm^2^ were eligible for transport studies.

#### 3.7.2. Transport Experiments

The Caco-2 cell membrane was washed twice with Hank’s buffer. Subsequently, 0.5 mL and 1.5 mL of fresh Hank’s buffer were added to the apical (AP) and basolateral (BL) sides, respectively. The cells were then cultured at 37 °C with 5% CO₂ for 30 min. The Hank’s buffer was then removed from both sides. A 0.5 mL solution of corn peptides (concentration 10 mg/mL, dissolved in Hank’s buffer) was added to the AP side, while 1.5 mL of fresh Hank’s buffer was added to the BL side. After 2 h of culture at 37 °C with 5% CO_2_, samples from the BL sides were collected, vacuum freeze-dried, and stored at −20 °C. The BL side sample contained corn peptides that were absorbed by the Caco-2 monolayer membrane.

### 3.8. Peptide Identification

Following desalting and freeze-drying, the corn peptide sample was dissolved in mobile phase A, filtered through a 0.22 μm filter membrane, and separated using the EASY-nLC 1200 ultra-high-performance liquid chromatography system. Mobile phase A comprised a 0.1% formic acid aqueous solution, while mobile phase B was a 0.1% formic acid and 80% acetonitrile aqueous solution. The liquid phase gradient was programmed as follows: from 0 to 80 min, the composition changed from 8% to 30% B; from 70 to 85 min, it transitioned from 30% to 40% B; and from 85 to 90 min, it reached 100% B, while maintaining a flow rate of 300 nL/min. Following separation by the ultra-high-performance liquid chromatography system, the peptide segments were ionized in the NSI ion source and subsequently analyzed using an orbitrap mass spectrometer. The ion source voltage was set to 2.8 kV, and both the precursor ions and their secondary fragments were detected and analyzed using the high-resolution Orbitrap. The primary mass spectrometry scan range was 200–2000 *m*/*z*, with a scan resolution of 70,000, and the secondary scan resolution was 17,500. The data acquisition mode employed a data-dependent scan (Full MS/DD-MS^2^) program. This program selected the top 20 peptides precursor ions with the highest signal intensity from the primary scan, sequentially subjected them to the HCD collision pool, fragmented using 27% collision energy, and then underwent secondary mass spectrometry analysis. To optimize mass spectrometry utilization, the automatic gain control (AGC) was set to 1E5, the signal threshold to 2E4 ions/s, the maximum injection time to 45 ms, and the dynamic exclusion time for tandem mass spectrometry scans to 15 s, reducing repeated scanning of precursor ions.

### 3.9. Molecular Docking

The crystal structure of yeast ADH (PDB ID: 5ENV) was obtained from the PDB database (http://www.rcsb.org/pdb/ accessed on 17 December 2023). The receptor protein pre-processing (deletion of water molecules and excess ligands, addition of hydrogen atoms) was accomplished using PyMOL 2.4 (Schrödinger Inc., New York, NY, USA). The two-dimensional and three-dimensional structures of the peptide were constructed using ChemDraw 19.0 and Chem 3D 19.0. The peptide and ADH were preprocessed using AutoDock Vina 1.1.2 for molecular docking. Docking boxes for receptors were identified using POCASA software and the relevant literature [21]. The dimensions of the protein ADH docking box were 58 Å × 52 Å × 40 Å with a grid spacing of 0.375 Å. The coordinates of the docking box were: x:y:z: −50.23:44.167:−22.274. The analysis of the molecular docking results from the molecular simulations provided the docking energy of the enzyme’s active site. The lowest docking energy corresponds to the most stable molecular docking structure. The visualization of the molecular docking results was conducted using PyMOL 2.4.

### 3.10. Synthesis of Peptides

The three purified peptides (purity > 98%) were synthesized by Qiangyao Biotechnology Co., Ltd. (Wuhan, China) using Fmoc solid-phase synthesis.

### 3.11. Statistical Analysis

All data were obtained from three parallel experiments, and the results were expressed as the mean ± standard deviation. The data were analyzed using SPSS 27.0.1 for one-way analysis of variance (ANOVA), and the Duncan multiple comparison test was employed for significance analysis. A *p*-value of less than 0.05 was considered to indicate a significant difference in the data.

## 4. Conclusions

In this study, the ADH activation rate was employed as the indicator to hydrolyze corn protein under optimal conditions using alkaline protease. The digestion and transport of corn ADH-activating peptides were investigated. By means of peptide sequence identification and molecular docking confirmation, three new peptides (SSNCQPF, TGCPVLQ, and QPQQPW) were identified that have the potential to activate ADH. All of these peptides could closely bind to the active center of ADH through multiple hydrogen bonds and hydrophobic interactions. The results of in vitro activity demonstrated that all three peptides exhibited varying degrees of the ADH activation. Among them, SSNCQPF exhibited the most notable ADH activation (EC_50_: 1.35 ± 0.22 mM). Therefore, it was demonstrated that corn gluten meal is an excellent source for the preparation of peptides with high activation ability to ADH, and the peptides released from corn protein are promising to intervene in ALD by activating ADH. Further research should focus on elucidating the in vivo effects of these peptides, exploring their pharmacokinetics, and assessing their therapeutic potential in ALD. Understanding the mechanisms underlying the activation of ADH by these peptides will be crucial for developing targeted interventions. Moreover, investigating the broader implications of these findings in the context of liver metabolism and related diseases could open new avenues for therapeutic strategies in ALD.

## Figures and Tables

**Table 1:** Peptide sequence from corn hydrolysates in both the control and BP side and binding energy with ADH.

No.	Peptide Sequence	Measured *m*/*z* (Da)	Charge	Calculated MW (Da)	Binding Energy (kcal/moL)
1	CENPILQ	408.6982	2	815.3847	−5.34
2	TGCPVLQ	717.3574	1	716.3527	−8.20
3	EVFEPF	767.3594	1	766.3538	0.95
4	SPFLGQ	648.3331	1	647.3279	−3.67
5	SSNCQPF	782.3066	1	781.3065	−8.69
6	DTPYSEF	429.6775	2	857.3443	−2.21
7	EVGDGVFE	426.1901	2	850.3709	1.26
8	TPYSEF	372.1648	2	742.3174	−3.42
9	FTPVLQ	704.3978	1	703.3905	−3.52
10	SVCENPAL	416.6947	2	831.3797	0.85
11	IFPQC	304.1461	2	606.2836	−5.65
12	TIFPQ	303.1659	2	604.3221	−3.49
13	QPQQPW	783.3767	1	782.3711	−7.31

**Table 2:** The detailed information of the three peptides combined with ADH.

Sequence	Binding Energy (kcal/moL)	Hydrogen Bonds	Hydrophobic Interaction	Salt Bridge	π-Stacking	π-Cation Interaction
SSNCQPF	−8.69	His 44 (3.55 Å), Gly 181 (3.56 Å), Leu 182 (3.11 Å), Gly 183 (3.63 Å), Val 245 (3.20 Å), Ser 246 (2.96 Å), Val 247 (3.56 Å, 3.72 Å), Met 270 (3.82 Å), Glu 333 (3.12 Å), Gly 339 (4.04 Å), Arg 340 (3.64 Å)	Phe 221 (3.62 Å, 3.90 Å), Val 247 (3.52 Å), Ala 251 (3.67 Å)	-	Phe 221 (3.78 Å)	-
QPQQPW	−7.31	His 44 (3.98 Å, 3.47 Å), Asp 53 (3.13 Å), Gly 181 (3.15 Å), Asp 201 (3.89 Å), Lys 206 (3.06 Å), Glu 333 (2.99 Å, 3.42 Å), Gly 335 (3.79 Å), Arg 340 (3.87 Å)	Leu 182 (3.84 Å, 3.58 Å, 3.58 Å), Phe 221 (3.27 Å), Val 247 (3.67 Å), Ile 337 (3.69 Å)	His 44 (4.31 Å, 5.14 Å)	-	His 44 (5.97 Å)
TGCPVLQ	−8.20	His 44 (2.80 Å, 3.15 Å), Thr 45 (3.64 Å), His 66 (3.45 Å), Thr 157 (2.72 Å), Gly 181 (3.85 Å), Asp 201 (3.09 Å), Lys 206 (4.06 Å), Val 245 (2.79 Å), Gly 335 (4.06 Å), Arg 340 (3.92 Å)	Thr 45 (3.89 Å), Trp 54 (3.61 Å), Trp 92 (3.69 Å), Leu 182 (3.78 Å), Phe 221 (3.57 Å), Val 245 (3.93 Å)	Lys 206 (5.09 Å)	-	-

Note: individual ‘-’ indicates no relevant receptor residues interacting with the peptide.

**Table 3:** ADH activation concentration for EC_50_ of synthetic peptides in vitro.

Sequence	ADH Activation EC_50_ (mM)
SSNCQPF	1.35 ± 0.22 ^b^
TGCPVLQ	2.26 ± 0.16 ^a^
QPQQPW	2.73 ± 0.13 ^a^

Note: different letters (^a^, ^b^) represent significant differences in values.

**Table 4:** Optimal enzymatic conditions.

Enzyme	pH	Temperature (°C)
Alcalase	8.5	60
Neutral	7.0	45
Flavourzyme	7.5	50
Protamex	7.0	55
Papain	8.0	50
Trypsin	8.0	37

## Data Availability

Data are contained within the article.
